# The Molecular Design of Active Sites in Nanoporous Materials for Sustainable Catalysis

**DOI:** 10.3390/molecules22122127

**Published:** 2017-12-02

**Authors:** Stephanie Chapman, Matthew E. Potter, Robert Raja

**Affiliations:** Department of Chemistry, University of Southampton, Highfield Campus, University Road, Southampton SO17 1BJ, UK; stephanie.chapman@soton.ac.uk (S.C.); M.E.Potter@soton.ac.uk (M.E.P.)

**Keywords:** Beckmann rearrangement, characterisation, structure-property correlations, zeotypes, acid sites

## Abstract

At the forefront of global development, the chemical industry is being confronted by a growing demand for products and services, but also the need to provide these in a manner that is sustainable in the long-term. In facing this challenge, the industry is being revolutionised by advances in catalysis that allow chemical transformations to be performed in a more efficient and economical manner. To this end, molecular design, facilitated by detailed theoretical and empirical studies, has played a pivotal role in creating highly-active and selective heterogeneous catalysts. In this review, the industrially-relevant Beckmann rearrangement is presented as an exemplar of how judicious characterisation and ab initio experiments can be used to understand and optimise nanoporous materials for sustainable catalysis.

## 1. Introduction

To meet the demands of an evolving global population, the chemical industry has expanded rapidly and, as a consequence, immense strain is being placed on both natural and synthetic resources. As a result of pressing environmental and economic concerns, the chemical industry has been driven towards more sustainable practices so that it can continue to meet the needs of the global populace.

The implementation of Green Chemistry is focal in the production of ubiquitous materials such as polymers and plastics, the precursors of which are predominantly derived from crude oil. Where processing chemical feedstocks to obtain higher-value products often involves many chemical transformations, catalysis has proven a widely-applicable and effective development. For example, many chemical processes invoke acid catalysis, and whilst mineral acids (such as hydrochloric and sulphuric acid) are accessible and cheap, their disposal, reuse and product purification are costly and detrimental to the environment. As a result, there has been an increasing shift towards the use of solid-acid catalysts that are more readily separated and recycled from fluid-phase reactions.

In this respect, the nylon industry is archetypal. As well as being the earliest synthetic polymers to be manufactured commercially, the polyamides (or nylons) have endured as some of the most industrially and economically important synthetic fibres in large-scale production [1]. Amongst the aliphatic polyamides, nylon-6 and nylon-6,6 are notable due to their widespread application in textiles and engineering resins; applications that exploit its characteristic elasticity, strength, and physicochemical stability. In accord with consumer demand, the nylon-6,6/-6 industry has been valued at $41.13 billion for 2025 [2].

**Scheme 1:**
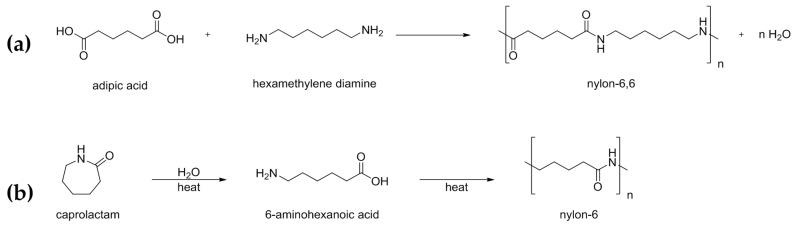
Scheme for the synthesis of the nylon-6,6 polymer through irreversible condensation reaction between adipic acid and hexamethylene diamine (**a**), and the nylon-6 polymer by ring-opening polymerisation of ε-caprolactam (**b**).

Typically, nylons are produced by an irreversible condensation reaction between a diamine and a dicarboxylic acid (Scheme 1a). Nylon-6, however, is formed by ring-opening polymerisation of the ε-caprolactam monomer (Scheme 1b). As a result, nylon-6 can be depolymerised and recycled, with >99% of the material recovered to make carpet yarn [3], a lifecycle far more compatible with the sustainable development agenda [4]. In contrast, its non-recyclable analogue, nylon-6,6, (which is formed by polymerisation condensation) can only be down-cycled for low-performance applications, and ultimately ends up in landfill sites. Nylon-6 dominates the global resin market (~60% total consumption in 2016) [2], and given its extensive application in textiles and engineering [5] (requiring 6 million tonnes in 2015) [6], reducing the negative environmental impact of the material lifecycle is hugely relevant. For this reason, there is significant interest, both in academia and industry, in developing novel heterogeneous catalysts that offer greater sustainability in the production of nylon-6.

**Scheme 2:**
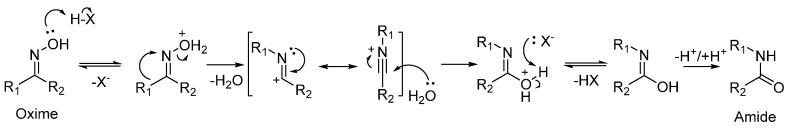
The general mechanism of the Beckmann rearrangement, in which the transformation of an oxime into an amide is facilitated by an acid catalyst (HX).

As well as being a fundamental precursor to nylon-6, ε-caprolactam is a high-value bulk chemical in its own right, with a global output expected to exceed 4.6 megatonnes by 2018 [7]. Typically, ε-caprolactam is produced from cyclohexanone oxime by the acid-catalysed Beckmann rearrangement. The Beckmann rearrangement is a classic, organic transformation in which an oxime is converted into an amide through acid catalysis (Scheme 2). Where a cyclic oxime substrate is used, a cyclic amide, or lactam, is formed.

**Scheme 3:**
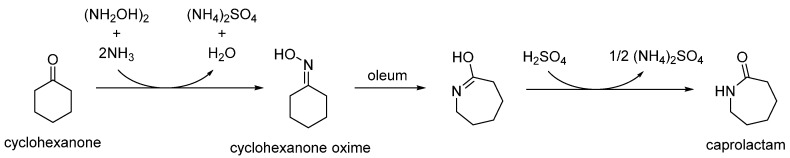
Conventional industrial route to ε-caprolactam using a homogeneous acid catalyst. In the first step, ammoximation of cyclohexanone yields cyclohexanone oxime, which then undergoes the Beckmann rearrangement to form ε-caprolactam (modified from reference [10]).

In the conventional, industrial route, this transformation is performed by a fuming sulphuric acid catalyst (oleum), in a multi-step process that generates disproportionate quantities of low-value ammonium sulphate by-product (Scheme 3) [8]. As this is both hazardous and inherently inefficient, conventional homogenous catalysts are being replaced by versatile solid-acid analogues. Besides their relatively facile recovery from fluid-phase reactions, heterogeneous catalysts also dispense with hazardous reagents and simultaneously alleviate the excessive amount of by-product generated by the traditional process [9].

One of the earliest industrial applications of a heterogeneous catalyst in the Beckmann rearrangement was reported in 1941 when “a practical and continuous method for carrying out the rearrangement of cyclohexanone oxime” using a silica gel catalyst was reported [11].

This concept was developed further, and in 1987 a heterogeneously-catalysed process that proved viable on a scale to rival conventional methodologies was implemented. In 2003, a plant in Niihama (Japan) employing an MFI-type catalyst was made operational, generating 65,000 tonnes of ε-caprolactam *p.a.* [6].

To date, the industrial Beckmann rearrangement remains predominantly a gas-phase process, using a fluidised bed reactor at >300 °C to maintain the solvent (typically ethanol) and both cyclohexanone oxime and caprolactam in the vapour phase. These conditions maximise catalytic activity (>98 mol % yield) and lifetime, whilst the continuous nature of the process eliminates catalyst separation steps. The success of the industrial vapour-phase process is also evident in the academic literature (Table 1), where many reports focus on solid-acid catalysts optimised for industrial, vapour-phase conditions. 

Several reviews deal comprehensively with the early literature pertaining to the commercial Beckmann rearrangement [24,25,26,27,28], but numerous innovations have been reported in the last decade. In particular, there has been a great deal of interest in developing a more energy-efficient, liquid-phase process that can operate below 150 °C. Whilst the vapour-phase Beckmann rearrangement is high-yielding, it is an energy-intensive process, and the high temperatures tend to promote the formation of unwanted by-products that lower reaction selectivity. Consequently, the development of an efficient, low-temperature process holds much promise, although the technology is currently in its infancy.

Following the success of the gas-phase process [26], considerable research has been devoted to understanding the properties of heterogeneous acid catalysts that impart a high activity and selectivity in the Beckmann rearrangement [27]. Whilst numerous materials have proven effective in catalysing the rearrangement, the prevalence of zeolites and related porous oxides (silicates, aluminophosphates, etc., referred to as ‘zeotypes’) is revealing. Such zeotype materials are used widely as solid-acid catalysts, particularly in industrial applications for which their particular physicochemical characteristics are well suited [27]. For example, inorganic porous oxides show excellent mechanochemical stability under a wide range of conditions that, in an industrial setting, translates into long catalytic lifetimes before regeneration is necessary. Most of these favourable attributes arise from their highly-crystalline microporous structures. Such regular porosity (on the scale of a small hydrocarbon molecule) places spatial constraints on molecular diffusion and hence product distribution. When the majority of the active sites are located internally (as opposed to on the surface or at the pore mouths) it is possible to moderate active site accessibility and thus direct a catalytic transformation. This fundamental property of zeotypes is the origin of their inherent reactant and product selectivity. Moreover, by placing the active site within the constrained environment of a microporous network, there is also the possibility of exploiting confinement effects to favour particular transition-states. Several zeolite structures (MFI, BEA, etc.), are known to provide a spatial fit that confers high selectivity in the vapour-phase Beckmann rearrangement [22,23,29]. Due to their potential for industrial implementation, there is significant interest in probing in detail the reaction kinetics and catalyst deactivation processes that operate in these zeolitic systems [30,31].

Whilst microporous zeotypes are renowned for their steric control of reaction mechanism, hierarchical analogues (that possess several levels of porosity) are also receiving considerable interest [32]. These materials have been shown to retain the favourable attributes of the microporous architecture (i.e., well-defined active sites and a good physicochemical stability), whilst simultaneously addressing mass transport limitations [33]. Thus, by incorporating an auxiliary mesoporous network, it is possible to enhance diffusion to and from the internal active sites, thus extending catalytic lifetime by retarding coke-induced deactivation [13,34].

**Figure 1:**
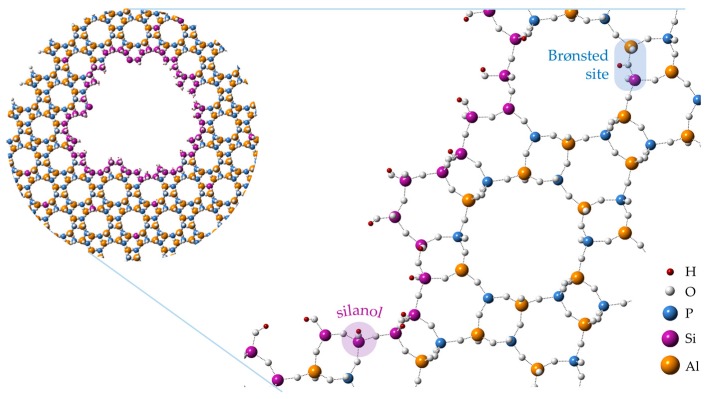
Depiction of hierarchical SAPO-5 prepared using an organosilane mesoporogen. The silanol sites are localised to the internal walls of the mesopore, whilst Brønsted acid sites are created in the bulk, microporous framework due to isomorphous substitution of silicon.

A common synthetic methodology for the production of hierarchical zeotypes involves the addition of a surfactant or oligomeric mesoporogen to the precursor gel [13,34,35]. This bottom-up, soft-templating route is appealing, as it is compatible with conventional one-pot syntheses and the organic mesoporogen is readily removed by the standard calcination protocol. As such, soft-templating has been employed in many instances for the production of hierarchical, zeotype Beckmann rearrangement catalysts. For instance, organosilane surfactants have been shown to integrate with silicoaluminophosphate (SAPO) frameworks so as to yield a homogenous hierarchical phase (Figure 1) [36]. Interestingly, in this case the production of mesopores was also accompanied by the formation of localised silanol sites, and the resulting hierarchical SAPO catalysts were found to sustain higher catalytic activity in the vapour-phase rearrangement than their microporous analogues.

Hierarchical SAPO-34 has also been prepared using a novel template system where surfactant encapsulated within MCM-41 acts as both Si-source and mesoporogen [12]. When tested in the vapour-phase Beckmann rearrangement of cyclohexanone oxime, the hierarchical SAPO-34 catalyst maintained high conversion and selectivity over 6 h on stream, whilst both microporous SAPO-34 and mesoporous MCM-41 showed lower activity and a marked degradation in performance throughout.

These results highlight the importance of combining appropriate acid-site characteristics with a favourable framework architecture in order to maximise catalyst efficacy. Another innovative method for creating mesoporous architectures has been presented by Palkovits *et al.*, who used oligomeric siloxane species to cross-link nano-sized particles of TS-1 [37]. The inter-particle voids generated by bridging TS-1 particles with amorphous silica were found to improve caprolactam yield under vapour-phase conditions, and also slowed catalyst deactivation. Inorganic templates have also proven to be effective in templating mesoporosity in zeotype Beckmann rearrangement catalysts. For example, Mg(OH)_2_ nanocrystals added to the synthesis gel yielded hierarchical MFI catalysts with modified textural characteristics. The resultant hierarchical framework showed improved mass transfer properties and enhanced vapour-phase activity compared to the conventional Silicalite-1 catalyst [38]. Beyond the MFI-type systems that are the usual target for vapour-phase Beckmann rearrangement catalysts, other promising systems have emerged. Among these is the microporous titanosilicate ETS-10, the hierarchical analogue of which has been produced, post-synthetically, using a microwave reactor [29]. The microexplosions produced by rapid degradation of hydrogen peroxide created larger pore structures in ETS-10 that boosted catalytic activity in the gas-phase Beckmann rearrangement.

As well as pore structure, the control of particle size is an important consideration in the synthesis of industrial vapour-phase catalysts. This type of manipulation can be achieved in a variety of ways, including modification of the ion content of the synthesis gel. In the synthesis of Silicalite-1, it has been shown that if the concentration of Na^+^ and Br^−^ exceeds 5 mol % (relative to the structure-directing agent) then larger particles are formed [39]. Despite possessing the same number of strong acid sites, the larger catalyst particles deactivated more rapidly due to greater coke deposition. Silicalite-1 particle size has also been modified via synthetic parameters such as ageing time and temperature, water quantity and lysine addition [21]. In this case, Silicalite-1 particles of ~50 nm exhibited optimal catalytic activity, selectivity and stability, as coke deposition at the pore mouth and particle surface was retarded. Alternatively, Kim *et al.*, used a novel linear structure-directing agent to create Silicalite-1 nano-sheets of 2 nm thickness. These nano-sheets displayed remarkable activity and lifetime, maintaining a high conversion and selectivity for over 10 times longer than conventional Silicalite-1 [22]. Additionally, seed crystals have been used to influence the particle growth of TS-1. Catalyst prepared from nano-sized (100–300 nm) precursor seeds showed greater activity after 6 h on stream than analogous TS-1 particles templated by micro-sized (4–6 μm) seeds [40]. The benefits of particle control are not confined to vapour-phase catalysts: nano-sized and delaminated zeotypes have also been exploited for the liquid-phase Beckmann rearrangement, where improved active site accessibility is especially advantageous in the transformation of the bulky cyclododecanone oxime substrate [41].

**Figure 2:**
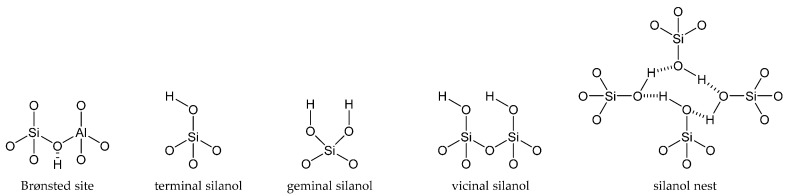
A depiction of the different types of acid site in zeotype materials (modified from reference [42]).

Framework architecture is seminal in zeolite science, and so too is the scope for active site design. Importantly, by combining both attributes appropriately, it is possible to synthesise a catalyst that is targeted towards a desired chemical transformation. For instance, some of the most applicable acid centres are silanol sites: neutral species with a small dipole moment in the Si-O bond that confers weak acidity. These sites are primarily found in zeolites (aluminosilicates) and pure silicates (such as SBA-15 and MCM-41) and are classed as terminal, geminal, vicinal or nest silanols, depending on the number of Si-O-Si bonds and their proximity to other silanol species (Figure 2) [42].

Intrinsically, silanols occur as framework defects, although their precise properties typically reflect the topology and Al/Si ratio of the material. In addition, there are a wide range of post-synthetic treatments that can be used to modify the type and concentration of silanols, and hence increase catalytic potential. For example, silylation of NbO_x_/SiO_2_ catalyst was found to improve ε-caprolactam selectivity and catalyst lifetime in the gas-phase Beckmann rearrangement. By passivating the more acidic, isolated-silanol sites with hexamethyldisilazane, the catalyst was more resistant to coking, and oxime hydrolysis, in the presence of water, could be suppressed [14]. Another common post-synthetic procedure involves the removal of framework atoms by treatment with an acid or base. Desilication by this route disrupts the crystalline microporous framework, creating disordered mesopores and defects as new silica surfaces are developed. When ZSM-5 catalysts of varying Si/Al ratio were subject to desilication under basic conditions, the resulting hierarchical zeolites exhibited improved yields of caprolactam in the liquid-phase Beckmann rearrangement due to improved mass transport capabilities. Whilst the synthetic methodology was found to remove a fraction of Brønsted sites, their acid strength was not modified by the procedure [43]. Equally, the selective removal of tetrahedral framework-aluminium (i.e., dealumination) creates silica nests that stabilise silicon atoms adjacent to the Al vacancy, although strong treatments can disrupt framework crystallinity. Ngamcharussrivichai *et al.* showed that a mild acid treatment could be used to enhance the activity of USY zeolite in the liquid-phase Beckmann rearrangement, whilst retaining framework crystallinity. Whilst dealumination was found to increase the concentration of favourable Brønsted acid sites, the creation of Lewis-acidic, extra-framework aluminium species was found to accelerate the formation of cyclohexanone side-product [44]. 

**Figure 3:**
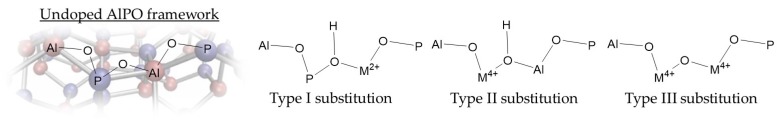
Three mechanisms of isomorphous substitution are observed for AlPO frameworks, depending on the formal charge of the dopant atom. M^2+/3+^ species tend to undergo Type I substitution of Al^3+^, whereas M^4+/5+^ preferentially replace P^5+^ by Type II. If a pair of M^4+^ species simultaneously substitute adjacent Al^3+^ and P^5+^ to create a M^4+^-O-M^4+^ bridge, this is designated a Type III substitution.

For commercial Beckmann-rearrangement catalysts, framework modifications focus predominantly on moderating acid or textural properties to improve catalytic activity and minimise coke deposits. In this context, Zhang *et al.* described the treatment of zeolite-β with aqueous ammonia solution to maintain a constant Si/Al ratio (and hence strong-acid site concentration), whilst increasing the amount of weak acid sites four-fold [23]. As a result, the NH_3_-modified zeolite-β showed a significant increase in catalytic lifetime, and achieved a caprolactam yield of 86 mol % after 8 h, an increase of 36 mol % over the standard zeolite-β system. In contrast, under liquid-phase conditions stronger acid sites are crucial if meaningful conversions are to be achieved. Purely-siliceous zeolites with only weak, external silanols are essentially inactive in the Beckmann rearrangement at 130 °C and instead promote oxime hydrolysis [45]. If stronger internal silanols (and the potential for substrate pre-activation by pore confinement) are introduced, moderate activity is observed under the same conditions. Finally, when Al is incorporated to form a zeolite framework, the resulting Brønsted acid sites create a vast improvement in both conversion of oxime and amide selectivity. Even wider scope for the catalytic application of zeotype materials been achieved by the isomorphous substitution of heteroatomic active sites. By doping into a crystalline network at low concentration, a distribution of well-defined, spatially-isolated active sites is created in what is described as a single-site heterogeneous catalyst (SSHC) [46]. By this route, a variety of metal dopants have been incorporated into zeotypes, each of which respond to the structural and chemical environment of the framework to create a unique active site. This phenomenon has been exploited in a wide range of zeotype species, perhaps most notably in the aluminophosphates (AlPOs). AlPOs are particularly amenable to isomorphous substitution (Figure 3), since the ionicity of the framework imparts flexibility to bond lengths and angles. Furthermore, AlPOs possess two markedly different T-sites (Al^3+^ and P^5+^), which offers additional scope for isomorphous substitution.

By this route, more than 25 main group and transition elements have been incorporated into AlPO frameworks [47]. In all cases, the formal oxidation state of the dopant heteroatom is the same, or lower than the framework atom it replaces. In the case that the formal charge of the dopant atom is less than the atom it replaces (e.g., Co^2+^ substituting Al^3+^, or Si^4+^ substituting P^5+^), the framework develops a net negative charge that must be balanced by a countercation. Typically, the cation is a framework-bound proton, with acidic properties that reflect the local atomic environment, namely the nature and location of the dopant atom, and the topology and composition of the framework. The Brønsted acid sites that arise through isomorphous substitution are typically much stronger than the aforementioned silanols; a characteristic that has been central in the development of catalysts for the Beckmann rearrangement. 

**Figure 4:**
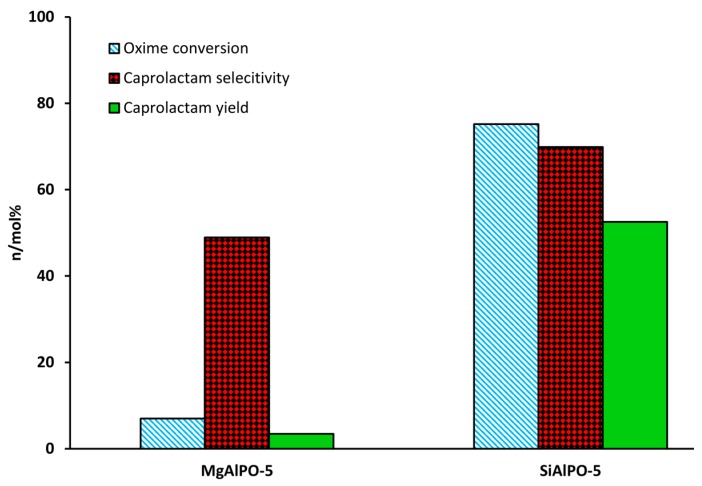
Activity of Mg- and Si-doped AlPO-5 in the vapour-phase Beckmann rearrangement of cyclohexanone oxime (WHSV 3.3 h^−1^, He carrier gas 20 mL min^−1^, 0.3 g catalyst, liquid feed 300 g L^−1^ of cyclohexanone oxime in methanol, 673 K) (modified from reference [48]).

Through adroit selection of the dopant species, it is possible to create targeted active sites, as is demonstrated in our own work. We found that by doping AlPO-5 with Mg^2+^ stronger acid sites are created—to the detriment of vapour-phase activity. In contrast, the Si^4+^-doped analogue (SAPO-5) contained significantly weaker acid sites that proved well-suited for catalysing the transformation (Figure 4) [48]. Significantly, by combining the strong acid sites from Mg^2+^-doping, with the weaker acid sites from Si^4+^-doping, it was possible to create a bimetallic system of intermediate acidity that showed improved caprolactam yield in the vapour-phase [20,49]. The aluminium-doped, mesoporous silica, Al-MCM-41, has also proven to be of great interest as a Beckmann rearrangement catalyst, since improved mass-transport behaviour has been linked to enhanced caprolactam selectivity and catalytic lifetime. Vaschetto *et al.* have demonstrated that, in the vapour-phase, the weak acidity of nest silanols endows a superior selectivity towards caprolactam [50]. On increasing the aluminium content of Al-MCM-41 (above a critical Si/Al = 60), these silicon nest sites were proliferated, with a concomitant increase in product yield [51]. Furthermore, by pre-treating the catalyst with linear alcohol (1-hexanol) the more acidic terminal silanol sites could be pacified as alkoxy groups, improving lactam selectivity further [18]. Quite contrastingly, in the liquid-phase, the surface silanol groups of mesoporous silicates are too weak to catalyse the Beckmann rearrangement. Thus, under mild reaction conditions, the activity of MCM-41, SBA-1 and SBA-15 could be enhanced by creating stronger acid sites through Al-doping [52]. Moreover, if Al was incorporated by post-synthetic grafting, the bound trigonal-chloride species yielded strong Lewis and Brønsted acid sites that showed better activity in the liquid-phase. Despite showing a high initial activity, the adsorption of caprolactam to the stronger acid sites of Al-grafted MCM-41 accelerated catalyst deactivation.

Not all elements are disposed towards isomorphous substitution as the framework often places restrictions on the size or charge of atoms that can be incorporated. Nonetheless, active species may otherwise be accommodated as extra-framework species. In fact, even those atoms eligible for framework substitution can be made to occupy extra-framework sites, provided an appropriate synthesis procedure is adopted. For example, in an effort to dope Zn^2+^ ions into AlPO-5, extra-framework species have been created [20,48]. Whilst, these extra-framework sites did not provide additional catalytic capabilities, their presence was found to modify the acidity of Mg- and Si-doped AlPO-5 systems antagonistically. Thus, bimetallic ZnSiAlPO-5 was found to possess stronger acid sites than SiAlPO-5, whilst MgZnAlPO-5 possessed weaker acid sites than MgAlPO-5. Therefore, extra-framework zinc species proved to be a valuable modifier that could be exploited to improve the yield of caprolactam obtained in MgZnAlPO-5 versus MgAlPO-5.

**Figure 5:**
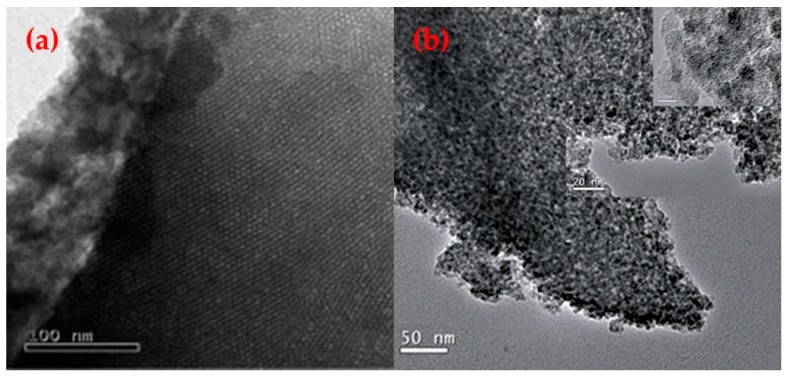
TEM images contrasting the mesoporous framework-substituted Al-MCM-41 species (**a**) with the extra-framework Nb sites in Nb/HMS-X (Si/Nb = 2) (**b**) (adapted from references [51,55]).

It is also possible to use post-synthetic methodologies to introduce non-metallic species into hexagonal mesoporous silicates (HMS). For example, using H_3_PO_4_ precursors, phosphorus has been successfully impregnated into the Al-MCM-41 framework [19]. After 7 h on-stream, the activity of the phosphorus-modified catalyst was found to exceed that of the parent Al-MCM-41 due to the combined effect of amplifying the number weak acid sites, whilst simultaneously reducing the quantity of stronger acid sites. Contrastingly, when boric acid was used to prepare an analogous boron-modified Al-MCM-41, catalytic activity in the vapour-phase rearrangement was attenuated by carbonaceous deposits, particularly at the strongest acid sites [53]. Under liquid-phase conditions, increasing the acid strength of Al-MCM-41 and Al-SBA-15 by applying a sulfonic acid (-SO_3_H) modification has proven to be beneficial to catalyst performance [54]. Nb/SiO_2_ systems containing extra-framework NbO_4_-tetrahedra have also proven to be highly effective catalysts for the Beckmann rearrangement [55,56,57,58]. TEM imaging has revealed the presence of extra-framework niobium sites at higher loading, with Nb-clustering favoured over framework substitution (Figure 5) [55].

When a small quantity of niobium (<0.3 wt %) is introduced on to a silica matrix, isolated NbO_4_ species are formed [55]. Whilst the resulting Nb/SiO_2_ system lacks the inherent Brønsted acidity typically sought in Beckmann rearrangement catalysts, such activity can be developed by reacting the Lewis-acidic Nb centres with ethanol, *in situ*. Raman and FTIR studies have shown that the interaction of Nb=O groups with co-solvent creates active NbO_3_(OEt)OH species that confer Brønsted acidity for the rearrangement [14,59].

In kinetic studies, both the adsorption energy of cyclohexanone oxime (154 kJ mol^−1^) and the activation energy for the Beckmann rearrangement (68 kJ mol^−1^) were found to be considerably lower than for the conventional Silicalite-1 catalyst [56]. Nb-doped materials have also proven active under liquid-phase conditions. A Nb-MCM-41 catalyst containing both tetrahedrally-coordinated framework Nb and extra-framework Nb_2_O_5_ species was used to catalyse both the ammoximation of cyclohexanone and subsequent Beckmann rearrangement in a one-pot reaction at 80 °C [60]. A higher Nb content was found to improve caprolactam yield by increasing catalyst acidity from weak to medium strength, however after optimising reactions conditions, only a 7.3 mol % conversion of cyclohexanone and 66 mol % selectivity for caprolactam was reported.

Indium, bismuth and tungsten species have also been grafted on to mesoporous silicas to induce catalytic activity. In the case of indium [16] and bismuth [17], it is proposed that the Lewis acidic metal interacts with the oxygen atoms of proximal silanol species, enhancing the acidity of the latter. In this case, improvements to catalytic activity and lifetime were found to be most prominent when the quantity of metal was very low (~3.5 wt %). However, in the case of WO_x_/SBA-15 [15], temperature-programmed desorption revealed that a higher quantity of WO_x_ yielded more medium-strength acid sites, which translated into improved catalytic performance and sustained activity on regeneration. 

It is the sheer scope for structural and chemical functionality in zeotype materials that make them excellent candidates for performing the acid-catalysed Beckmann rearrangement. Fundamentally, these materials offer a robust, porous framework with tuneable acid characteristics, although designing an effective solid-acid catalyst requires an understanding of the nature of the active site, the catalytic pathway and the role of the host. Correspondingly, the literature pertaining to the characterisation of catalysts for the Beckmann rearrangement is extensive. Whilst a host of spectroscopic techniques have been exploited in order to study catalyst structure and mechanism, *in situ* and *in operando* techniques in particular have provided opportunities to probe the interactions between active sites and reactive species in a more representative chemical environment. These studies emphasise the importance of comprehensively evaluating the structure-property relationships that elicit catalyst activity, and highlight the profound link between catalyst performance and a controlled catalytic environment.

## 2. Vibrational Spectroscopy

In the development of heterogeneous catalysts for the Beckmann rearrangement, considerable research has been devoted to optimising framework acidity to maximise caprolactam yield. Effective catalysts bind the oxime substrate with sufficient affinity to promote the desired transformation, yet not so strongly as to retain the lactam product. As a result, acid-site characterisation has become inextricably linked with catalyst design, as is illustrated by infrared (IR) spectroscopy.

IR is a well-established vibrational technique that is widely employed in catalysis for both materials characterisation and *in situ* studies. A range of IR technologies are available, making it an inclusive and informative technique. Commonly, IR spectroscopy is performed in transmission-mode, whereby infrared radiation is propagated through a sample whilst the frequency of resonant absorption of vibrational modes is monitored. However, where transmission IR is incompatible with a (for example, strongly-absorbing) sample, a reflectance protocol may be adopted. Such techniques include Diffuse Reflectance Infrared Fourier Transform Spectroscopy (DRIFTS) and Attenuated Total Reflectance Infrared Spectroscopy (ATR-IR); surface-sensitive methods that are suited to studying chemistry occurring at the interfaces. The wide applicability of the IR technique has led to its integration as part of routine analysis for ascertaining the nature, quantity and strength of the acid sites in a framework, and from this, structure-property relationships have been extrapolated. In addition, by employing more advanced methodologies, such as probe-based techniques or combinatorial studies (e.g., DRIFTS-XAS) it is possible to monitor synchronous changes in the catalyst and surface species under *in situ* or *in operando* conditions.

Unsurprisingly, in a review of the literature pertaining to catalysts for the Beckmann rearrangement, IR studies are pervasive. In its simplest form, IR spectroscopy is used to characterise framework acidity based on hydroxyl stretching modes in the 3000–3800 cm^−1^ range. In this region, the precise position of an O-H stretching band is informative of the acid site characteristics, being sensitive to local variations in bond length and angle, and the electrostatic influence of the coordination environment. For the same reason, infrared spectra also exhibit a topological dependence that can be used to distinguish the same type of active site in different framework locations (Table 2). For example, microporous SAPO-34 exhibits two characteristic O-H stretching bands (at 3633 and 3610 cm^−1^) that have been attributed to two types of Al(OH)Si Brønsted sites [36]. Here, the band at 3633 cm^−1^ arises from a proton attached to the O(4) framework oxygen (which resides in one four-membered and two eight-membered rings), whilst the peak at 3610 cm^−1^ corresponds to a proton bond to the O(2) oxygen (which resides in one four-membered, one six-membered and one eight-membered ring) [47].

The nature of high-yielding active sites in solid-acid, Beckmann rearrangement catalysts has been the subject of much debate. The consensus is that (unlike the industrial homogeneous system) in the vapour-phase, production of caprolactam is favoured when a heterogeneous catalyst is only very weakly acidic. Evidence to this effect has largely arisen from the concerted effort to characterise and optimise the industrially-favoured, high-silica MFI zeolite catalyst.

The hydroxyl stretching region of the H-ZSM-5 zeolite, which has been well-characterised by infrared spectroscopy [62], exhibits two key bands. An absorbance at ~3720 cm^−1^ indicates terminal hydroxyls, identified by ammonia-TPD as weaker acid sites, that are supposedly derived from surface termination and framework defects. The other peak at 3600 cm^−1^ is a characteristic framework mode of the MFI zeolite and arises due to the presence of Brønsted acid sites. As is typical of zeolites [63], the stretching frequency of this mode varies linearly with the Si/Al ratio [64], and hence framework acidity [65].

**Figure 6:**
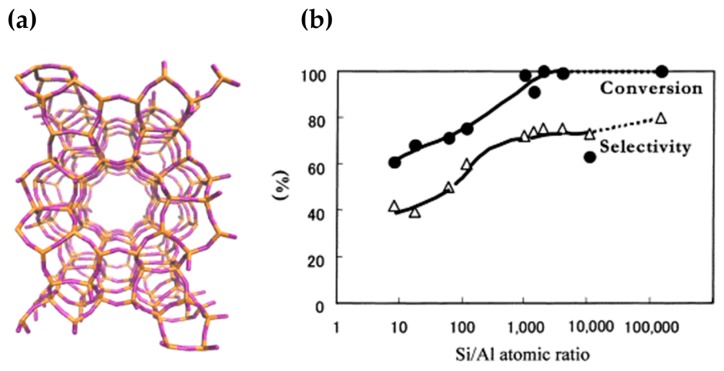
The MFI structure of Silicalite-1 (**a**), and the influence of Si/Al ratio on the conversion of cyclohexanone oxime (•) and selectivity to caprolactam (▵). Reaction conditions: cyclohexanone oxime (8 wt %) in benzene solution, WHSV = 3 h^−1^, 623 K (**b**) [68].

When a range of MFI framework constitutions were studied for activity in the Beckmann rearrangement, it was found that caprolactam yield was maximised using high-silica MFI catalysts (Figure 6). In their study, Sato *et al.* noted that exceptional catalytic activity was obtained at Si/Al > 2000, when the strongest Brønsted acid sites were no longer detectable by ammonia-TPD. Even pure-silica MFI (Silicalite-1) was found to catalyse the Beckmann rearrangement with 100 mol % conversion and 80 mol % selectivity for ε-caprolactam. Thus, it was concluded that the Brønsted sites arising from Al incorporation were not the active sites for the vapour-phase Beckmann rearrangement, rather it was the very weakly-acidic, or otherwise neutral silanol sites that were responsible for this activity [66,67].

In a combined spectroscopic-catalytic investigation of the active sites in siliceous acid catalysts, Hölderich *et al.* were able to characterise the silanol sites of the MFI catalyst that were responsible for high activity in the Beckmann rearrangement [69]. Using FTIR spectroscopy, three types of silanol site were observed: terminal (3740 cm^−1^), vicinal (3690 cm^−1^) and nest (3500 cm^−1^), with acidity decreasing in that order, whereas crystalline Silicalite-1 presented only terminal sites, its higher-yielding, ammonia-treated analogue [70], possessed additional vicinal and nest silanol groups. These weaker silanol sites were considered more effective catalytically, as their appearance was accompanied by a marked increase in lactam yield. The results also vindicated previous reports that by purposefully removing silanol groups in H-ZSM-5 catalysts using trimethylchlorosilane, lactam selectivity could be improved from 85 to 95 mol % [71]. In this case, IR analysis of the modified catalyst showed a significant decrease in the intensity of the silanol peaks, particularly for the stronger terminal sites, whilst a C-H stretching mode 2970 cm^−1^ was evolved, confirming the methyl-capping. For the same reason, the industrial high-silica MFI is exposed to methanol at high temperature, since capping the more reactive (and acidic) terminal silanols as an alkoxy group improves selectivity for caprolactam [26,68].

IR in its native form is not a quantitative technique. The half-width at half-maximum of a vibration is modified by inhomogeneity in the local environment of the active site (such as framework defects or hydrogen-bonding interactions), as well as anharmonicity in the vibration [72]. Therefore, to perform a quantifiable assessment of an acid site, a knowledge of the extinction coefficient of the corresponding vibrational mode is required. As this is not always practicable, especially for solid-acid systems, IR can be made into a quantitative technique by coupling with probe-based studies [72].

In probe-based FTIR, a catalyst is exposed to a gaseous, basic molecule that interacts with the acid sites in a manner that ranges from hydrogen bonding interactions through to protonation. Suitable probe molecules form a well-known adduct, with characteristic bands in largely unpopulated regions of the IR spectrum [73]. Typically the extinction coefficient of a characteristic probe vibration is known, allowing peak area to be correlated with the concentration of the adsorbed species. Through acid-base interactions, it is possible to assess the strength of the acid site by the degree of red-shift of the framework O-H mode (Δν_OH_), since stronger acid sites translated to a greater degree by interaction with a probe molecule. It is also possible to quantify active site concentration through the decay of framework bands and/or the growth of characteristic modes of the sorbed probe. Moreover, by monitoring the IR spectrum of the sorbate-adsorbent system over a range of temperatures, the sorptive properties of the probe molecule can be used to determine acid-site strength [74]. In this sense, probe-based IR mimics temperature-programmed desorption (TPD) studies, except that desorption of the basic molecule is monitored through the loss of characteristic vibrational modes, rather than by measured desorption.

Depending on the nature of the probe molecule, targeted study of acid characteristics is possible [12]. Carbon monoxide is the most widely used probe molecule for zeotype species, as it can readily diffuse through the smallest pores, accessing a wide range of acid sites. Where the frequency of the C≡O stretch is sensitive to the nature and strength of the acid site, it is possible to distinguish different acid sites based on ν(CO) (Table 2). However, CO is a weakly-basic molecule and as such, adsorption will only occur at acid sites of sufficient strength to maintain an interaction. In contrast, NH_3_, a much stronger base, will also interact with weaker acid sites. By employing larger probe molecules (such as pyridine, lutidine or collidine), it is possible to approximate the location of the acid sites within a framework. Due to their larger kinetic diameter, bulky probe molecules will only interact with acid sites that are made accessible by the pore structure and the proximity of other available active sites. Thus, it is common practice to use a range of probe molecules to obtain a more comprehensive understanding of the location and strength of the acid sites. By extension, if the probe molecule is also the reactive substrate, it is possible to monitor, *in situ*, the active sites that participate in catalysis and hence determine catalyst mechanism. Such was the basis of an *in situ* FTIR study by Flego and Dalloro, who investigated the activity of weak silanol nest sites in the Beckmann rearrangement [74]. By tracking the adsorption and transformation of cyclohexanone oxime on Silicalite-1 using FTIR, the authors found direct evidence for the involvement of silanol active sites in the transformation to lactam, and used this to establish a reaction mechanism. Fundamentally, the interaction of multiple hydroxyl groups within a silanol nest orients the oxime substrate in a reactive conformation that preserves cyclic geometry and stabilises the intermediate species. In contrast, the more acidic, isolated silanols were found to strongly adsorb both the oxime and lactam, to the detriment of reaction selectivity.

**Figure 7:**
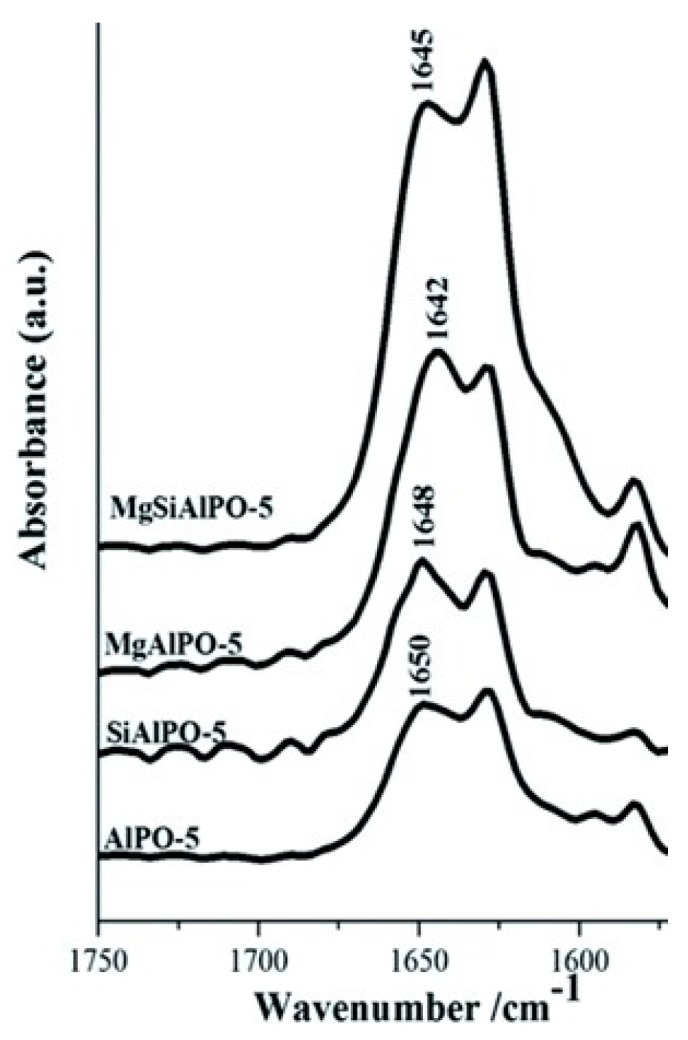
The FTIR spectra of 2,6-dimethylpyridine dosed on AlPO-5 and heteroatom-substituted analogues, shown at 298 K and after degassing. The relative peak position of the *ν*_8a_ mode (the labelled peak in the 1655–1640 cm^−1^ range) reveals the intermediate strength of MgSiAlPO-5, since a lower wavenumber correlates with stronger Brønsted acid sites (modified from reference [49]).

Given the compelling evidence in favour of weak acid sites for the vapour-phase Beckmann rearrangement, heteroatom-doped AlPOs have since been investigated. These materials allow single-sites to be engineered with desirable characteristics through the choice of framework dopant and pore architecture [42]. Whilst there is immense scope for modulating the acid characteristics of heteroatom-substituted AlPOs to target the Beckmann rearrangement, the importance of dopant identity has been illustrated in our earlier work comparing the performance of Mg^2+^ and Si^4+^-doped AlPO-5 (AFI) [48]. Using carbon monoxide and 2,6-dimethylpyridine in probe-based FTIR studies, it was possible to correlate the poor yield of caprolactam obtained using MgAlPO-5 with the presence of strong acid sites. It was proposed that these stronger sites retain the lactam product more strongly, impairing selectivity. In contrast, SiAlPO-5, which was found to possess a greater proportion of weaker acid sites, gave superior performance in vapour-phase process. More recently, Gianotti *et al.* studied the effect of dual-substitution of Mg and Si [49]. The resultant bimetallic MgSiAlPO-5 material, which had an acidity intermediate of the monometallic systems (Figure 7), exhibited synergistic enhancements in catalytic activity, with improved yield of caprolactam. These results highlighted further scope for modulating active site strength in AlPOs for optimal activity in targeted transformations. In general, SAPOs have proven well-suited for catalysing the vapour-phase Beckmann rearrangement on account of their weak, tuneable acidity [12,36,75]. Recently, hierarchically-porous (HP) SAPOs have been engineered to combine the desirable physicochemical characteristics of the microporous material with an auxiliary mesoporous network in order to enhance diffusion and catalyst longevity. In our previous work, Newland *et al.* describe soft-templating to introduce mesoporosity into microporous SAPO frameworks [36,76]. The FTIR technique (amongst others) was used to characterise Brønsted sites (3628–3600 cm^−1^) arising from the isomorphous substitution of Si into HP SAPO-34 and HP SAPO-5 frameworks. In addition, the vibrational spectra of these catalysts revealed a band at 3746 cm^−1^, which was absent in their microporous analogues. This mode was attributed to pendant silanol sites within the mesopores, originating from the siliceous surfactant template. It was proposed that the interplay between the weaker silanol sites and the isolated Brønsted centres was responsible for the enhanced catalytic performance of the hierarchical catalysts in the Beckmann rearrangement.

Hierarchical SAPO-34 has also been synthesised using surfactant encapsulated within MCM-41 as a combined mesoporogen and silicon source [12]. To investigate the nature, strength, and accessibility of the Brønsted acid sites, the hierarchical material was subject to probe-based FTIR. Due to the weak basicity of CO and its ability to permeate the small pores of the framework, the molecule can differentiate acid sites of different strength. In the FTIR spectra of CO adsorbed at 80 K, the hydroxyl stretching of the Brønsted acid sites (3630 and 3610 cm^−1^) in HP SAPO-34 and SAPO-34 was red-shifted by hydrogen bonding to CO, forming an intense absorption centred at ~3350 cm^−1^. The extent of this red-shift was found to be comparable in both microporous and hierarchical SAPO-34, indicating that their Brønsted acid sites were of similar strength. Similarly, the corresponding C≡O stretching mode was blue-shifted by 32 cm^−1^ through interaction with Brønsted acid sites in both CHA materials. Additionally, for HP SAPO-34 it was possible to differentiate the acid sites within the micropores from those in the mesopores by varying the kinetic diameter of the basic probe. Whilst the sorption of ammonia, like CO, is relatively unhindered, its stronger basicity makes it less discriminative in its interaction with acid sites. Hence, NH_3_ was used to quantify the total framework acidity. In contrast, bulky substituted-pyridines (with kinetic diameters in excess of the micropore dimensions) can only interact with the acid sites that are accessible within the mesopores or at the micropore mouths. Illustratively, the O-H stretch of the Brønsted acid sites in HP SAPO-34 were found to only partially diminish on exposure to 2,4,6-trimethylpyridine (collidine) vapour, suggesting that only a fraction of these sites were accessible to the molecules. In fact, using probe-based FTIR it was possible to quantify that <4 % total Brønsted acid sites in HP SAPO-34 were available to the substituted pyridines. Nonetheless, the improved accessibility of the acid sites in HP SAPO-34 resulted in significant enhancement in the yield of ε-caprolactam versus SAPO-34, MCM-41, or a mechanical mixture of these phases. Thus, it was emphasised that these bimodal catalysts integrate improved mass transfer with the favourable acid-site characteristics of the microporous framework.

SAPO-37, a silicoaluminophosphate with the FAU-type structure has been shown particularly effective in catalysing the liquid-phase Beckmann rearrangement. In SAPO materials, Si can be incorporated into the framework by Type II or Type III isomorphous substitution mechanisms, leading to the formation of isolated Brønsted sites (Type II), or silicon islands (Type II and Type III) [77]. Due to a preference for incorporating Si via the Type II mechanism [78], SAPO-37 contains a largely homogeneous distribution of isolated Brønsted acid sites. This can be advantageous in developing targeted, solid-acid catalysts, since such isolated Brønsted sites are weaker than those produced by silicon islanding. Thus, by controlling the fraction of acid sites generated by Type II substitution, it is possible to tune the acid characteristics of SAPO-37. On this basis, we developed a SAPO-37 catalyst that achieved near-quantitative yield of ε-caprolactam in the liquid-phase Beckmann rearrangement [79,80]. However, it is noteworthy that other systems containing weakly-acidic sites (e.g., MCM-41) show poor activity in the liquid-phase reaction, indicating that (unlike the vapour phase) a certain strength of acid site is required. Using a range of characterisation tools (TPD, solid state NMR, INS and FTIR), we studied the effect of Si-loading on the acid characteristics SAPO-37. Due to a preference for site-isolation, at low Si-loading SAPO-37 contained predominantly weak, isolated Brønsted sites. However, on increasing dopant levels, silicon islanding became increasingly prevalent, leading to the formation of stronger acid sites, as revealed by NH_3_-TPD. Significantly, selectivity towards the desired lactam product was found to correlate with weaker acidity, and hence lower Si-loadings [80].

**Figure 8:**
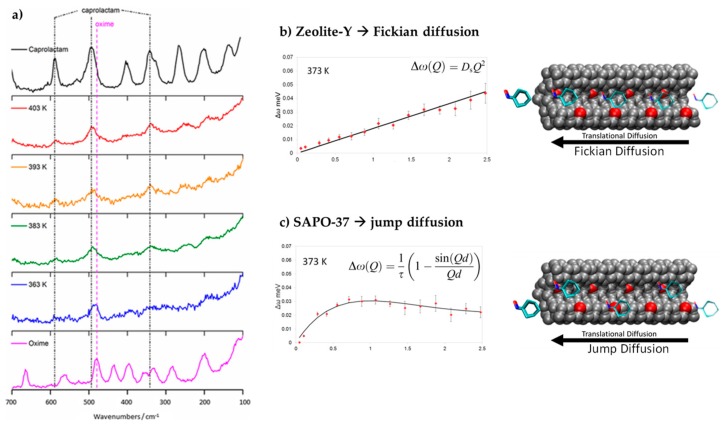
In a physical mixture of SAPO-37 and cyclohexanone oxime, INS studies revealed that ε-caprolactam was formed significantly below the reaction temperature of 130 °C (**a**) [80]. QENS studies revealed that cyclohexanone oxime, in a physical mixture with Zeolite-Y (FAU), exhibits smooth, Fickian diffusion (**b**), whereas in SAPO-37, the oxime is found to undergo jump diffusion due to favourable interaction with the internal acid sites (**c**) (modified from references [79,80]).

In order to probe the activity of SAPO-37 in the liquid-phase Beckmann rearrangement, the interaction of cyclohexanone oxime with the framework was examined [79]. In this case, the less conventional vibrational technique of inelastic neutron spectroscopy (INS) was employed. Whilst vibrational spectroscopy is a natural choice for the characterisation of surface-bound adsorbate, the sensitivity of optical techniques is often limited by the opacity of the zeolite and an inability to access informative, low-energy modes in the fingerprint region. However, in INS, the SAPO framework is effectively transparent because hydrogen atoms are significantly more effective at scattering neutrons [81]. Thus, INS spectra are dominated by the hydrogenous modes of key catalytic moieties (such as framework hydroxyls and silanols) and reactant species. Remarkably, the INS studies revealed that in a solvent-free environment, caprolactam was formed significantly below the reaction temperature, rationalising the efficacy of the isolated acid sites in SAPO-37 for the liquid-phase Beckmann process (Figure 8). A different neutron technique, quasi-elastic neutron scattering (QENS), was used to study the diffusion of cyclohexanone oxime in SAPO-37 [79]. At 373 K, cyclohexanone oxime, in a physical mixture with SAPO-37 catalyst, was found to undergo jump diffusion, with a jump distance of 4.5 Å, corresponding to the separation between the faujasitic supercages. Such behaviour contrasts with Zeolite-Y (FAU), which exhibits smooth, Fickian diffusion of the oxime under the same conditions. We attributed the jump diffusion behaviour to interaction between the oxime and the internal acid sites that facilitate the Beckmann rearrangement. This was corroborated by INS, which revealed concomitant loss of vibrational modes of both the framework hydroxyls and oxime O-H groups when the two species were intermixed. These spectroscopic studies reveal differing interactions between cyclohexanone oxime and the weak acid sites of the SAPO-37 framework, versus the stronger sites in its zeolitic analogue. It is the favourable, low-temperature interactions of the Brønsted sites in SAPO-37 that were deemed responsible for the high selectivity towards caprolactam in the Beckmann rearrangement.

## 3. Solid-State Nuclear Magnetic Resonance (ssNMR) Spectroscopy

With the development of new technologies such as superconducting magnets and specialised pulse sequences, solid-state (ss)NMR has become an increasingly informative and applicable technique in catalyst characterisation [82,83,84]. Moreover, increased compatibility with a range experimental conditions has provided greater scope for *in situ* studies of catalyst mechanism, as has been actively demonstrated for the Beckmann rearrangement.

Due to the ascendancy of zeotype materials in heterogeneous catalysis, some nuclei are observed more routinely by ssNMR than others. For the purpose of structural characterisation, ^29^Si-NMR is common. The number and type of silanol species within a catalyst can be determined using 1D ^29^Si magic angle spinning (MAS) NMR, however 2D and cross-polarisation (CP) MAS NMR are also used to resolve discrete environments by exploiting the sensitivity of Si nuclei to neighbouring spin-active nuclei [80]. Common ^29^Si signals are summarised in Table 3. For purely siliceous frameworks, it is typical to quantify the Q^3^ species as these identify with the active sites for the vapour-phase Beckmann rearrangement.

Depending on framework constitution, ^27^Al, ^31^P and ^11^B nuclei may also be probed using ssNMR. ^27^Al-NMR is less sensitive than ^29^Si-NMR and as such offers relatively limited information, although it is possible to quantify *intra*- and *extra*-framework aluminium species based on the intensity of AlO_4_ (59 ± 2 ppm) and AlO_6_ (0 ± 2 ppm) resonances, respectively [87]. In some cases the penta-coordinate aluminium of surface species may be resolved, however this is challenging without the use of 2D MAS NMR. Similarly, ^31^P-NMR can be used to establish a tetrahedral oxide environment (as in AlPOs), which presents itself as a peak at around −30 ppm [80,88]. NMR analysis of ^11^B nuclei is more definitive of the structural chemistry of boron-doped systems, differentiating the nucleus as it alternates between tetrahedral coordination (B(OSi)_4_, −4 ppm) in a hydrated system, and trigonal (5–10 ppm) when dehydrated [89]. Furthermore, boron chemical shift is sensitive to the local atomic environment: a study of B-Ge-ZSM-5 was able to resolve the local environment of boron through a B(OSi)_4_ signal at −4.1 ppm, B(OSi)_3_(OGe) at −2.9 ppm, and B(OSi)_2_(OGe)_2_ at −1.7 ppm [90].

**Figure 9:**
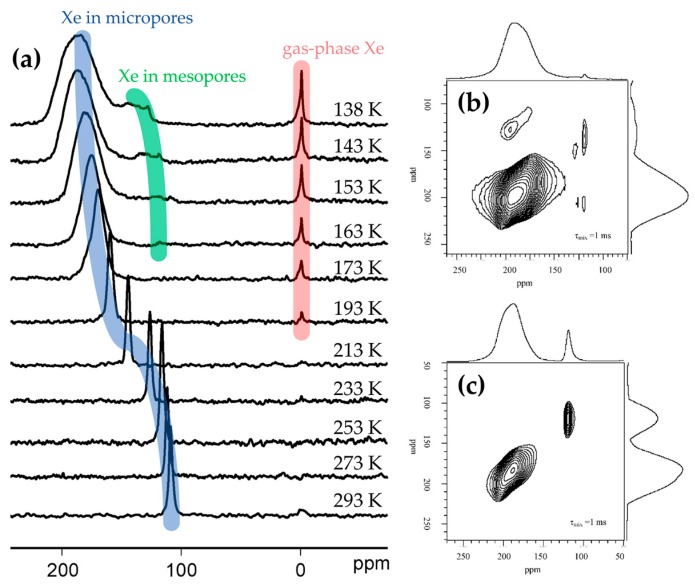
^129^Xe-NMR of Meso-ZSM-5 (50) between 293 and 138 K, showing the presence of Xe with different spatial constraints (**a**). Laser-hyperpolarised ^129^Xe 2D-EXSY NMR with a mixing time of 1 ms of Meso-ZSM-5 (50) at 143 K (**b**). Off-diagonal signals are due to Xe transferring between micropores (δ ~ 200 ppm) and mesopores (δ ~ 120 ppm). Laser hyperpolarised ^129^Xe 2D-EXSY NMR of a physical mixture of microporous ZSM-5 and mesoporous silica at 143 K, with a mixing time of 1 ms (**c**). A lack of off-diagonal peaks indicates slower diffusion of Xe between the micropores (δ ~ 200 ppm) and mesopores (δ ~ 120 ppm) (modified from reference [92]).

Solid-state NMR has also been used to study the porosity of framework materials via ^129^Xe-NMR [91,92,93]. In zeotypes, the chemical shift of a ^129^Xe probe molecule is sensitive to both surface interactions and also the uniformity and connectivity of the pores. By controlling the temperature and pressure of ^129^Xe adsorption, the textural characteristics of the framework can be established. Moreover, using hyperpolarised 2D exchange NMR spectroscopy (EXSY) on hierarchically-porous systems it is possible to measure the rate at which Xe interchanges between the micropores and mesopores, and hence assess diffusion and pore connectivity (Figure 9) [92]. This technique has been used to investigate how post-synthetic treatment of Silicalite-1 affects catalytic activity in the vapour-phase Beckmann rearrangement [93]. In this case, ion exchange and calcination protocols were found to modify the hydroxyl population of Silicalite-1. Specifically, low-pH treatment and higher calcination temperature was found to favour geminal silanol sites, leading to reduced catalyst activity and lifetime. ^129^Xe-NMR chemical shift showed that Xe was interacting less effectively with the pore wall where the higher concentration of Si(OH)_2_ defects created more space in the pores. Thus, the authors attributed the enhanced catalyst degeneration to enhanced coke formation in the larger pores [93]. To directly probe the acid sites in zeotype materials ^1^H-MAS-NMR may be employed [89,94,95]. Using this technique it has been possible to distinguish Brønsted acid protons (4.0 ppm) and silanol species (1.8 ppm) in mordenite zeolites [96]. However, assigning bands can be challenging, especially where multiple proton environments exist in a single system. Moreover, the chemical shift of a particular acid site will vary between frameworks as the local environment of the proton changes. In analogy with probe-based FTIR, it is also possible to characterise acid sites by their interaction with a probe molecule, by tracking the ^1^H-NMR signal as pressure or temperature is varied. For example, boron-substituted ZSM-5 (H-[B]ZSM-5) was subject to the adsorption of basic probe molecules and studied by ^1^H- and ^11^B-MAS-NMR spectroscopy [89]. In the proton spectrum of the dehydrated H-[B]ZSM-5, three signals due to silanol groups (1.9, 2.5 and 3.2 ppm) were identified. However, on adsorption of ammonia and pyridine the signal at 2.5 ppm was diminished, allowing the assignment of acidic bridging-hydroxyl groups adjacent to a framework boron atom (SiO(H)B). Where ssNMR revealed that the proton affinity of H-[B]ZSM-5 was greater than its aluminosilicate analogue, it was possible to establish the weaker acidity of SiO(H)B groups versus the SiO(H)Al Brønsted sites of the zeolite.

**Scheme 4:**
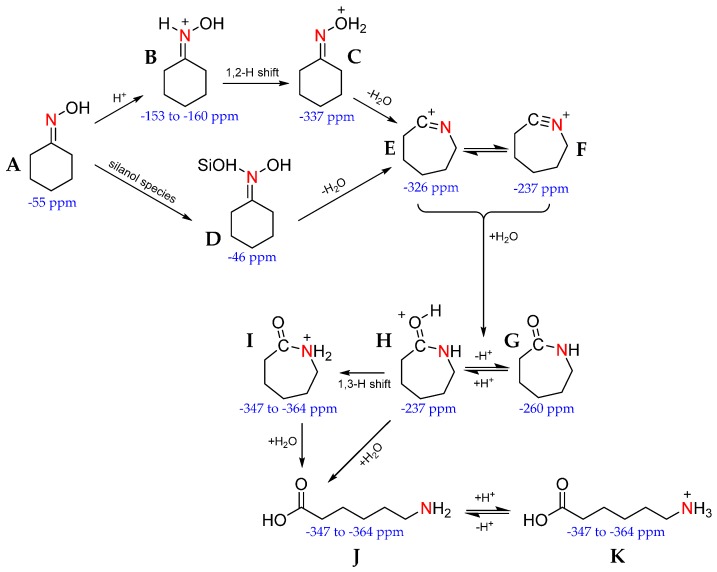
The mechanism of the Beckmann rearrangement, determined experimentally using ^15^N-MAS-NMR on Silicalite-1 and H-ZSM-5 and ^15^N-labelled cyclohexanone oxime. The numbers in blue indicate the ^15^N chemical shifts assigned using DFT modelling (modified from references [98,99]).

The technique of ^1^H-ssNMR has also been extended to *in situ* studies of substrate-framework interactions between cyclohexanone oxime and zeolites. In one such study, the proton environment of Silicalite-1 (MFI, Si/Al = 1700) was contrasted with that of ZSM-5 (MFI, Si/Al = 14) [94]. When Silicalite-1 was loaded with cyclohexanone oxime, a resonance due to hydrogen-bonded oxime was identified at 9.3 ppm. On heating, the intensity of this peak was reduced, with concurrent growth of a signal at 12.2 ppm due to hydrogen-bonded caprolactam. In contrast, the more acidic ZSM-5 framework (with acidic bridging hydroxyls groups producing a signal at 4.2 ppm), showed both hydrogen-bonded (9.3 ppm) and protonated oxime (11.2 and 13.0 ppm). Subsequent heating of the ZSM-5 system led to a strong decrease in the signals at 11.2 and 13.0 ppm, whilst a peak at 14.2 ppm was evolved due to the formation of protonated caprolactam. Furthermore, ε-aminocaproic acid by-product (identified by the signal of the NH_2_ group at ~6.5 ppm) was detected in the presence of ZSM-5, due to ring-opening of ε-caprolactam by the stronger acid sites. MAS-NMR studies of isotopically labelled oxime substrate have proven to be invaluable in establishing the mechanism of the vapour-phase Beckmann rearrangement (Scheme 4) [97,98,99]. Using ^1^H/^13^C and ^1^H/^15^N cross-polarisation MAS NMR, Fernández *et al.* probed the mechanism of the Beckmann rearrangement of isotopically-labelled acetophenone oxime in (siliceous) beta-D and (Al-containing) H-beta-D zeolites [95]. The studies revealed two reaction pathways for the rearrangement, depending on the strength of the acid site. Whilst both silanol and Brønsted sites were catalytically active, the acidity of the silanol groups was insufficient to protonate acetophenone below the transformation temperature (473 K), instead forming hydrogen-bonded complexes. In contrast, the Brønsted sites present in aluminium-doped, H-beta-D zeolite were able to effect *N*-protonation at room temperature.

Further insight has been gained by comparing the signals of weakly acidic and neutral catalysts (SBA-15 and Silicalite-1, respectively) with those containing Brønsted acid sites (ZSM-5) to correlate acid-site strength with mechanism pathway. Where weakly acidic species are unable to protonate the cyclohexanone oxime (**A**) to form the *N*-protonated oxime (**B**), reaction does not proceed via the 1,2-H shift to the *O-*protonated oxime (**C**). Instead, cyclohexanone oxime forms a hydrogen-bonded adduct with several silanol groups (**D**) that is transformed to the nitrilium ion (**F**). Equally, weak and neutral sites are unable to protonate the caprolactam product (**G**), instead the lactam is physically adsorbed to SiOH sites. In contrast, stronger acid sites tend to produce *O*-protonated (**H**) and *N*-protonated (**I**) lactam product. In the presence of water, this can drive ring-opening reactions to form by-products, such as ε-aminocaproic acid (**J** & **K**). When these experiments were performed in the presence of ^13^C-labelled methanol, the alcohol solvent was found to react with the strong acid sites to form ethers, alkenes and, more importantly, water, through dehydration reactions. The water was then available to react with the protonated caprolactam, to the detriment of reaction selectivity [98,99].

Presently, there remains ample opportunity to perform analogous ssNMR studies on the liquid-phase Beckmann rearrangement, where there is considerable evidence to suggest that the mechanistic pathway deviates from that of the vapour phase. As ssNMR technology continues to improve, a wider range of experimental conditions are becoming accessible, and the use of isotopically-labelled species is allowing greater insights into molecular transformations. For example, ^17^O-NMR, in conjunction with ^1^H-NMR (via HETCOR, MQMAS, REDOR and CP techniques), has emerged as a powerful technique for probing oxygen sites. Whilst this technique has not yet been applied to study the Beckmann rearrangement, there is great potential in using ^17^O-NMR to assess parameters such as bond length, acid site location, proton exchange rate, etc. [96]. Conceivably, NMR experiments could also be used to improve our understanding of the catalyst deactivation pathways that affect the Beckmann rearrangement, as has already been demonstrated for the methanol-to-olefin process.

## 4. Computational Insights

Undeniably, innovations in spectroscopic analysis have driven numerous advances in the field of catalysis, both in active site design and mechanistic studies. In tandem with this, an exponential growth in computing power and *ab initio* methods has made it possible to decipher complex catalytic phenomena occurring at an atomic-level. Significantly, by integrating theoretical and empirical studies it has become possible to both predict and rationalise macroscale events at a fundamental level.

In the literature, computational studies relating to the Beckmann rearrangement overwhelmingly employ either quantum mechanical (QM) or density functional theory (DFT) methods, with relatively few examples of molecular dynamics (MD) or simulations. However, within the sphere of theoretical science, there are two foci: mechanistic studies (calculating the energetics of intermediates and transition states, in both gaseous media and within confined pores) and the interpretation of characterisation data (calculating chemical properties of reaction intermediates and assigning spectral signals). Other areas of study, such as kinetic modelling or catalyst deactivation, are reported far more infrequently for the Beckmann rearrangement.

Initially, QM calculations for the acid-catalysed Beckmann rearrangement used gas-phase species (i.e., single, isolated molecules with no confinement effects or host framework) to corroborate the four key steps of the experimentally-derived mechanism: *N*-protonation of the oxime, 1,2 hydride-shift, rearrangement and tautomerisation. Other studies performed under these conditions have offered additional insights into the Beckmann process, for example how aprotic solvents promote by-product formation through hydrolysis of oxime starting material [100], or how the presence of water lowers the activation energy of the rate-determining step (RDS) [101]. Whilst insightful, these early gas-phase studies tended to over-estimate activation energies to approximately double that determined experimentally (~200 kJ mol^−1^ compared to ~100 kJ mol^−1^) for zeotype systems [101]. Such values are typically obtained by specifying a reaction-coordinate (such as a bond length) and constructing a reaction profile. By measuring the energy of the transition state (relative to the starting material) over a range of reaction coordinate values, it is possible to deduce the structure of species that lie close in energy to the transition state (the structure with typically the highest energy in the reaction coordinate). By then applying a transition-state optimisation to ensure a single, imaginary vibrational-frequency contibution, a more accurate description of the transition state and the activation energy can be determined. More advanced methodologies can perform transition-state optimisation using only the coordinates of the reactant and product species, however the better the initial guess, the more realistic the outcome [102].

**Figure 10:**
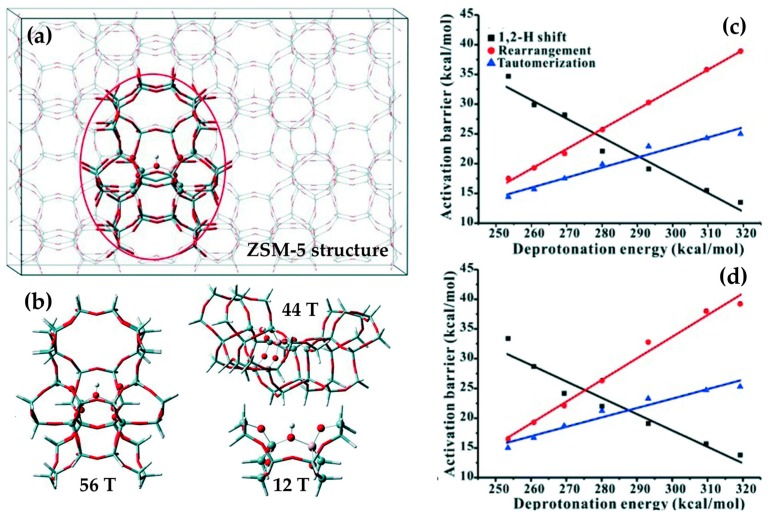
The cluster used for a 56 T model of ZSM-5 (**a**). The different cluster-sizes used to evaluate the influence of spatial constraints (**b**). The influence of acid-site strength on the energetics of the Beckmann rearrangement of cyclohexanone oxime (**c**) and for acetoxime (**d**) (modified from references [107,108]).

More recently, cluster models have been used to study the catalytic sites (including Brønsted sites, silanol nests and isolated silanols) that are active for the Beckmann rearrangement (Figure 10) [103,104,105]. In the case of zeotype materials, cluster models typically invoke a ring of 10–12 T-sites, depending on the framework used, and smaller oximes (formaldehyde oxime or acetoxime) to minimise the computational cost [101,103,106]. Using this approach, the activation energy for each mechanistic step of the Beckmann rearrangement was assessed for the different types of acid site [105]. The multi-step model showed that, depending on the active centre, the energetics of the reaction could be modified to such an extent that the rate-determining step (RDS) was altered. In this case, different strength acid sites show variable degrees of interaction with reactive species, causing the energetics of transition states (and hence the RDS) of the Beckmann rearrangement to be modified. This was particularly noticeable for the rearrangement of cyclohexanone oxime in mordenite zeolite, where stronger Brønsted acid sites were more effective at stabilising the transition state for nitrogen-insertion relative to weaker silanol species [105]. As a result, at a Brønsted site the activation energy for nitrogen-insertion step fell below that of carbiminium ion hydrolysis, whereas at a silanol site nitrogen-insertion remained the largest activation barrier [105]. Although insightful, this type of ‘bare cluster’ model still shows discord with experimental activation energies. The methodology has, however, proven well-suited to the study of homogeneous catalysts, where there is little need for periodicity or confinement.

To account for confinement effects in porous systems, two computational models have been developed. One option is to model a larger cluster (up to 72 T-sites) or a periodic framework, although this is a relatively computationally-demanding approach [107,108]. Alternatively, a combined QM/MM method can be adopted, whereby the active site and oxime are modelled using QM, whilst the influence of the wider framework is accounted for using MM force fields [106,109]. This is known as the ‘embedded-cluster’ approach. In either case, by including confinement effects it has been shown that the Van der Waals (VdW) interactions between cyclohexanone oxime and zeotype pore walls leads to stabilisation of the transition states. As such, embedded-cluster models have achieved a harmony between theoretical and experimental results.

More recently, computational techniques have been used to extrapolate the relationship between acid-site strength and reaction energetics. For weakly-acidic microporous zeotypes (e.g., B-ZSM-5), a higher deprotonation energy (273–319 kcal mol^−1^) was found to correspond to a well-stabilised transition state for the 1,2-H shift (activation energy 14 kcal mol^−1^), relative to tautomerisation and rearrangement (25 and 38 kcal mol^−1^, respectively). However, on increasing the acid site strength (i.e., deprotonation energy <273 kcal mol^−1^) transition-state of the 1,2-H shift is destabilised, whilst that of the tautomerisation and rearrangement steps is stabilised (35, 15 and 18 kcal mol^−1^, respectively). A linear correlation between acid-site strength and activation energy indicated that a system of moderate acidity (i.e., a deprotonation energy of 275–285 kcal mol^−1^) would exhibit the net lowest activation energy for the process [107]. However, both experiment and computation have shown that the same phenomenon is not active in the weakly-acidic, mesoporous MCM-41 system, where the larger pores (and hence weaker VdWs interactions) are unable to stabilise the transition states to the same extent [107]. Computational modelling has also proven instrumental in locating the active site of the industrial MFI catalyst; a controverted topic in the Beckmann rearrangement literature. Based on empirical studies, it was proposed that small pore apertures of the MFI framework (~5.4 Å) hinder the migration of caprolactam through the framework, confining the Beckmann rearrangement of cyclohexanone oxime to external sites on the catalyst surface. Nonetheless, adsorption studies also showed that both cyclohexanone oxime and caprolactam could be internalised by Silicalite-1, suggesting that the rearrangement process could occur at internal acid sites within the micropores. Based on computational arguments, however, it has since been proposed that catalysis occurs at nest silanols, located just inside the pore mouths of the MFI framework [108]. In this study, the energetics of the transition states associated with the 1,2-H shift, rearrangement, and tautomerisation steps at 598 K were compared for a surface and inside a pore. It was shown that, whilst VdW interactions offset the enthalpic contributions associated with confining cyclohexanone oxime within the MFI pore, a high entropic barrier favoured reaction at the pore-mouths or external surfaces [108].

Arguably, it is the integration of theory with characterisation that has been authoritative in catalyst studies, as demonstrated by solid-state NMR studies [110]. When boron-doped zeolite was combined with isotopically-labelled ^15^N-cyclohexanone oxime, a host of signals were produced in both the ^11^B- and ^15^N-MAS-NMR spectra. However, using DFT modelling it was possible to calculate the chemical shift of various intermediate and bound structures, and hence assign the NMR signals [111]. In this way, it was possible to elucidate the mechanism of the Beckmann rearrangement in B-ZSM-5 and B-Zeolite-β. Similar protocols have also been employed in the wider remit of solid-acid catalysis, illustrating, for example, how the ^1^H-NMR chemical shift of a bound pyrrole molecule can be used to determine the acid strength of zeolites [112].

Theoretical studies have provided invaluable insight into catalyst mechanism and directed material design. Nonetheless, in a comprehensive understanding of catalyst operation, many areas are still available for novel *ab initio* studies. Molecular mechanics simulation, for example, has much to offer, particularly with the emergence of hierarchical species. Such simulations could be used to model the diffusion pathways of reagents and products under realistic operating conditions in order to assess how pore structure affects mass transport. As *in operando* and *in situ* techniques become more mainstream, the potential for synchronising these with theoretical studies offers further opportunities. *Ergo*, it will be important to develop more holistic computational models that can extend beyond the fundamental substrate-framework interactions to incorporate other important experimental parameters, thus evolving model structures into realistic systems.

## 5. Conclusions

As the chemical industry strives towards more sustainable operations, large-scale production of ε-caprolactam is being revolutionised by the development of highly-selective solid-acid catalysts. These advancements have been underpinned by advancing characterisation techniques and computational methods that seek to understand catalyst activity and mechanism through the influence of zeolite structure and interactions. Studies of the Beckmann rearrangement process in nanoporous materials has demonstrated the power of allying theoretical and empirical studies. The active-site design that has been achieved through the combination of techniques described in this venture serves as a valuable blueprint to the catalysis community as a whole.

## Figures and Tables

**Table 1:** An overview of heterogeneous catalysts with reported activity in the vapour-phase Beckmann rearrangement.

Catalyst	Temperature/°C	WHSV/hr^−1^	Solvent	Conversion/mol % ^a^	Selectivity/mol % ^b^	TOS/hr ^c^	Reference
HP-SAPO-34	325	0.79	ethanol	99	95	6	[12]
SAPO-34	325	0.79	ethanol	63	92	6	
MCM-41	325	0.79	ethanol	15	92	6	
HP-S-1 acid	350	1.5	ethanol	61	95	60	[13]
HP-S-1 calc	350	1.5	ethanol	58	52	60	
S-1	350	1.5	ethanol	25	48	20	
Nb_2_O_5_/SiO_2_	400	17.1	ethanol	99	95	26	[14]
WO_x_/SBA-15	350	0.6	methanol	75	92	10	[15]
In/SBA-15	350	1.2	benzene	98	97	6	[16]
Bi/SBA-15	350	1.2	benzene	100	100	1	[17]
SBA-15	350	1.2	benzene	64	79	1	
Al/MCM-41	350	2.8	1-hexanol	76	100	60	[18]
Al/MCM-41	420	0.44	ethanol	93	70	7	[19]
P/Al/MCM-41-3	420	0.44	ethanol	97	83	7	
MgAlPO-5	400	3.3	methanol	7	49	1	[20]
SiAlPO-5	400	3.3	methanol	75	66	1	
MgSiAlPO-5	400	3.3	methanol	12	43	1	
S-1 (210 nm)	370	6	ethanol	100	88	4	[21]
S-1 (120 nm)	370	6	ethanol	99	94	4	
S-1 (56 nm)	370	6	ethanol	100	96	4	
MFI nanosheet	350	3	ethanol	69	92	80	[22]
MFI Bulk	350	3	ethanol	18	7	20	
H-Beta	350	0.95	methanol	57	87	8	[23]
H-Beta-NH_3_	350	0.95	methanol	96	90	8	

^a^ Conversion of cyclohexanone oxime, ^b^ selectivity to ε-caprolactam, ^c^ time on stream.

**Table 2:** Assignment of the FTIR hydroxyl stretching modes (ν(OH)) of SAPO materials and their shift (Δν(OH)) following CO adsorption. The activity of the SAPO catalysts in the vapour-phase Beckmann rearrangement is also reported.

Material	Site	ν(OH)/cm^−1^	ν(OH··CO)/cm^−1^	Δν(OH)/cm^−1^	Catalytic Activity in the Vapour-Phase Beckmann Rearrangement at 1 h		
					Conversion of Cyclohexanone Oxime/mol %	Selectivity for Caprolactam/mol %	Reaction Conditions
SAPO-37 [61]	Al–OH/P–OH	3680	-	-	>99	86	300 °C, WHSV 0.3 hr^−1^, He flow 33.3 mL min^−1^
	Si(OH)Al	3641					
	Si(OH)Al	3575	-	-			
SAPO-34 [36]	Si-OH	3746	-	-	92	70	325 °C, WHSV 0.79 hr^−1^, He flow 33.3 mL min^−1^
	Al–OH/P𢀓OH	3678	-	-			
	Si(OH)Al	3633	3347	286			
	Si(OH)Al	3610	3281	329			
SAPO-5 [36]	Si-OH	3746	-	-	71	76	325 °C, WHSV 0.79 hr^−1^, He flow 33.3 mL min^−1^
	Al–OH/P–OH	3678	3368	270			
	Si(OH)Al	3638	3369	268			

**Table 3:** Typical ^29^Si-NMR assignments for zeotype Beckmann rearrangement catalysts.

Material Type	Species			Signal/ppm
Silicates [85]	Si(OSi)_4_	Q^4^	Tetrahedral framework silica.	110
	(HO)Si(OSi)_3_	Q^3^	Terminal, nests or bridged silanol.	103
	(HO)_2_Si(OSi)_2_	Q^2^	Geminal silanol.	90
Silicon-substituted aluminophosphates [80]	Si(OSi)_3_(OAl)	T^4^	Framework silica with an adjacent Al atom.	108
	Si(OSi)_2_(OAl)_2_	T^4^	Framework silica with two adjacent Al atoms.	104
	Si(OSi)(OAl)_3_	T^4^	Framework silica with three adjacent Al atoms, typically at the periphery of a silicon island.	101
	Si(OAl)_4_	T^4^	Isolated silicon atom, leading to a Brønsted acid site.	92
Boron-substituted zeolites [86]	Si(OSi)_3_(OB)	Q^4^	Tetrahedral silicon with adjacent boron atom.	110
